# Do animal models of brain tumors replicate human peritumoral edema? a systematic literature search

**DOI:** 10.1007/s11060-023-04246-1

**Published:** 2023-02-09

**Authors:** Moritz W. J. Schramm, Stuart Currie, Ming-te Lee, Laurent J. Livermore, Sandeep P. Solanki, Ryan K. Mathew, Heiko Wurdak, Mihaela Lorger, Chris Twelves, Susan C. Short, Aruna Chakrabarty, Paul Chumas

**Affiliations:** 1 Leeds Teaching Hospitals NHS Trust, University of Leeds, Leeds, UK; 2 University Hospital Coventry & Warwickshire NHS Trust, Coventry, UK; 3 School of Medicine, University of Leeds, Leeds, UK; 4 Department of Neurosurgery, The General Infirmary at Leeds, Great George Street, Leeds, LS1 3EX UK

**Keywords:** Animal model, Brain tumor, Edema

## Abstract

**Introduction:**

Brain tumors cause morbidity and mortality in part through peritumoral brain edema. The current main treatment for peritumoral brain edema are corticosteroids. Due to the increased recognition of their side-effect profile, there is growing interest in finding alternatives to steroids but there is little formal study of animal models of peritumoral brain edema. This study aims to summarize the available literature.

**Methods:**

A systematic search was undertaken of 5 literature databases (Medline, Embase, CINAHL, PubMed and the Cochrane Library). The generic strategy was to search for various terms associated with “brain tumors”, “brain edema” and “animal models”.

**Results:**

We identified 603 reports, of which 112 were identified as relevant for full text analysis that studied 114 peritumoral brain edema animal models. We found significant heterogeneity in the species and strain of tumor-bearing animals, tumor implantation method and edema assessment. Most models did not produce appreciable brain edema and did not test for observable manifestations thereof.

**Conclusion:**

No animal model currently exists that enable the investigation of novel candidates for the treatment of peritumoral brain edema. With current interest in alternative treatments for peritumoral brain edema, there is an unmet need for clinically relevant animal models.

## Introduction

Peritumoral brain edema is a key contributor to morbidity and mortality in brain tumors resulting in mass effect and raised intracranial pressure [1, 2].

Brain edema is broadly divided into fluid accumulation within cells (cytotoxic edema) or in the interstitial space (vasogenic edema) [3], although the two usually coexist to a greater or lesser extent [4]. Intracellular or “cytotoxic” edema is thought to arise from disordered metabolism (e.g. as a consequence of ischaemia) inside cells allowing fluid to enter the cells [4]. By contrast, extracellular or “vasogenic” edema is thought to arise from dysfunction in the Starling forces that govern the passive exchange ingress and egress of fluid between the vasculature and interstitial space [4]. In intrinsic high-grade, metastatic brain tumors and some meningiomas extracellular vasogenic edema is usually the major contributor and is believed to result from leakiness of the blood–brain barrier (BBB), driven by factors such as abnormal neovascularization, and changes at the subcellular level with disrupted tight junctions, fenestrations of endothelia, increasing pinocytic vesicles and abnormalities of the basal membrane [5]. At the molecular level, vascular endothelial growth factor (VEGF) and inflammatory cytokines such as leukotriene C4, nitrous oxide (NO) and prostaglandin E2 have been implicated.

Dexamethasone, a synthetic corticosteroid, has been used to control peritumoral brain edema since the 1950s [6, 7] and up to a fifth of patients with a malignant brain tumor take steroids for the remainder of their lives (23.3 weeks) from the time of diagnosis [8]. However, corticosteroids have significant adverse effects that increase over time as the cumulative dose increases [8–11]. In practice, it can be very challenging to balance the therapeutic and adverse effects of corticosteroids; indeed, recent studies suggest that corticosteroids may actually increase mortality in patients with GBM [12, 13]. There is, therefore, increasing interest in finding alternative agents for the control of peritumoral brain edema. Accordingly, various candidates have been investigated in animal models as potential alternatives to dexamethasone [13–15].

Since their first use in the late 1940s, numerous categories of animal model have arisen, each with their own strengths and weaknesses [16]. These animal models can be broadly divided according to the host animals’ species (e.g. mouse, rat, cat etc.) and strain (e.g. Fischer vs BDIX vs Sprague–Dawley rats). They can also be categorized according to the method used to generate tumors in the host animals, which include chemical induction of tumors and implantation/injection of established tumor cell lines, and implantation/injection of xenogeneic implants of either established cell lines or patient-derived tissue in immunodeficient animals. In primary brain cancer models, cancer cells are injected orthotopically into the brain. This same method is frequently used to study macrometastases in the brain. However, hematogenous brain metastasis models, where cancer cells are administered intracardiac or into the internal carotid artery and colonize the brain from the blood stream, better recapitulate metastatic disease [17, 18]. More recently, genetically engineered mouse models which reproducibly develop intracranial tumors and humanized mouse models allowing transplantation of human tumors into partially immunocompetent animals have been developed [16, 19]. Understandably, in developing these models, investigators have sought greater levels of fidelity in replicating the histopathological and genetic makeup of human tumors in animals; replicating the effects of the resulting tumor on the surrounding normal brain tissues, including the development of intratumoral brain edema, may not have been a design priority.

When testing new agents for the treatment of peritumoral brain edema, pre-clinical models should be carefully assessed regarding their suitability for this task. We here present a systematic scoping review of different pre-clinical brain tumor models claiming to study peritumoral brain edema. As steroids are most commonly necessary for long periods of time in the treatment of primary and secondary tumours within the brain parenchyma, where radiotherapy is a frequent adjunct and surgery not always possible, we limit the review to studies modelling such tumours. This deliberately excludes meningiomata, where peritumoral edema does occur, but is a more variable phenomenon.

## Methods

### Literature search

A systematic search was undertaken of 5 literature databases (Medline, Embase, CINAHL, PubMed and the Cochrane Library). No limits were set for date of publication or language. The generic strategy was to search for various terms associated with “intracranial tumors”, “brain edema” and “animal models”. The detailed search strategy for each database can be found in Appendix A.

### Report selection

Reports were screened in two passes. First, two reviewers (MWJS and SPS) screened report titles and clearly unrelated reports were eliminated. Subsequently, two reviewers (MWJS and M-T L) screened the abstract of the remaining reports and eliminated any remaining reports that did not meet the inclusion criteria and those in non-English languages. The inclusion criteria were any form of animal model and any form of intracranial tumor induction method (e.g. injection/implantation of tumor cells, genetically-induced tumors, chemically-induced tumors) was deemed sufficiently relevant to proceed to data extraction. Disagreements at both stages were resolved by discussion. The remaining reports underwent full text review and data were extracted by a single author (MWJS). Data were extracted for each animal model – some reports studied more than one – and included: host animal characteristics (species; strain; immunity status), tumor characteristics (induction method; cell line, where relevant; syn- or xenogeneity), and reported measures of edema (e.g. histopathology; neuroimaging; brain water content (BWC) etc.).

### Imaging review

**Fig. 1:**
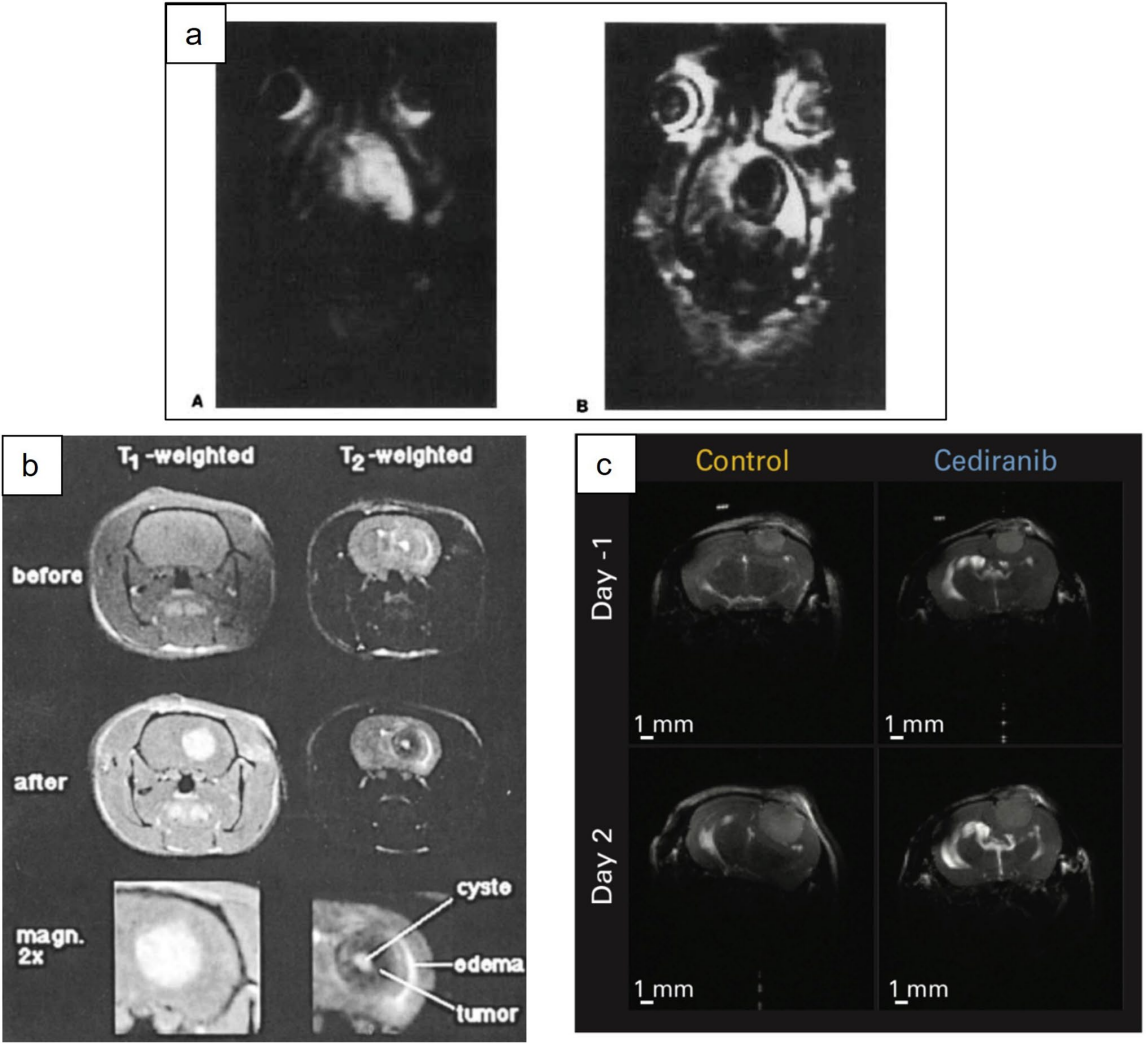
Representative MR images of different edema patterns obtained in animal models. **a** an example of infiltrative tumor edema captured on T1 IR snapshot FLASH imaging from rats bearing F98 glioma, reproduced with permission[52]. **b** an example of a peritumoral ‘halo’ of edema, demonstrated in T1- and T2-weighted MR images in Fischer rats bearing F98 glioma, reproduced with permission[30]. **c** an example of tumor with no detectable edema on T2-weighted MR in nude mice bearing U87 tumors, reproduced with permission [25]

The subset of selected reports which showed MRI sequences of peritumoral brain edema were reviewed separately. Of twenty-eight reviewed articles, four were excluded as they were devoid of MR imaging. Of the remaining 24 research papers 16 were rat models, 4 were mouse, 3 were cat, and 1 was dog. MR images were reviewed by a consultant neuroradiologist (SC) and assigned the presence or absence of peritumoral edema. Cases with peritumoral edema were classified in to one of two patterns, either ‘halo’, i.e. a rim of edema around the tumor or ‘infiltrative’ i.e. edema extending along white matter tracts and also classified by extent depending on whether the volume of edema was volume less, or greater than the volume of the tumor volume, see Fig. 1.

## Results

**Fig. 2:**
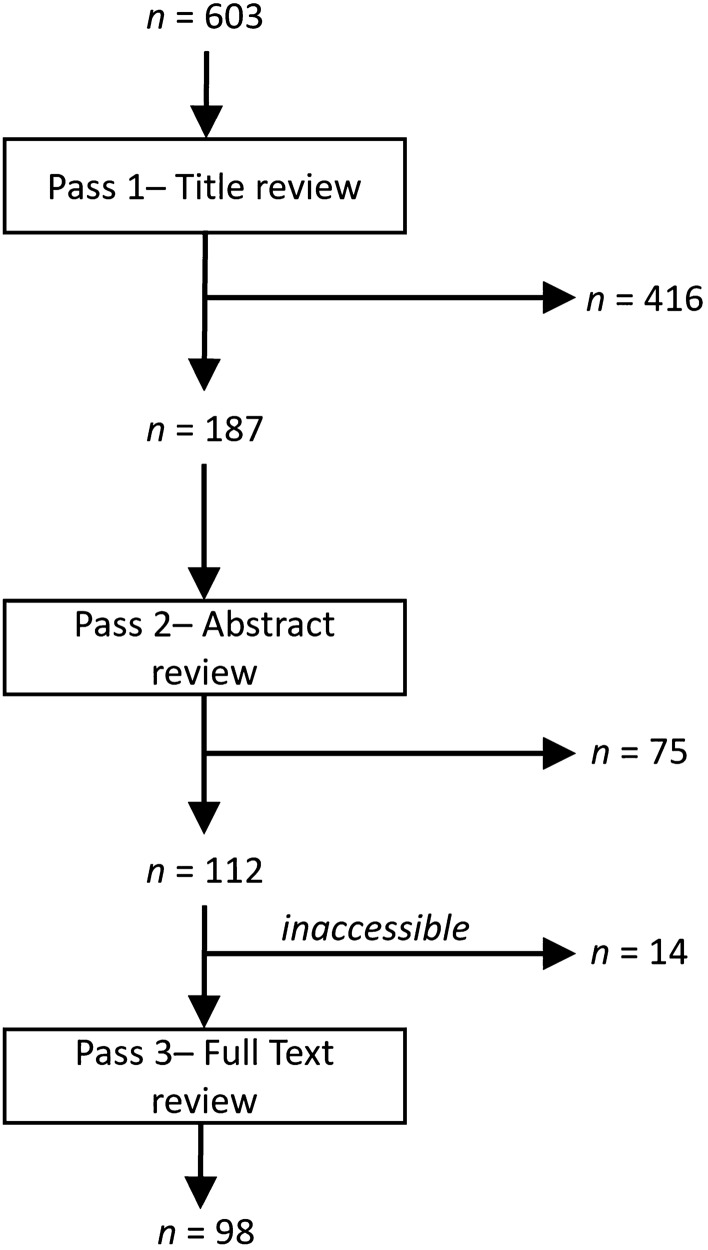
Report selection process

Our search identified 603 reports which were narrowed down to 112 for full text review; 14 reports could not be accessed (Fig. 2) due to our library lacking access. Some of the remaining 98 reports alluded to more than one animal model, so the final number of animal models under evaluation (114) was greater than the number of reports. 53 studies involved the assessment of a particular anti-edema agent and were therefore specifically designed to assess peritumoral edema.

We report our findings in two separate ways. First, we will take a high-level view of the type of model used (see Table 1). We then progressively break down the proportion of animal models by host species, the host strain and the method of tumor generation. The most common animal model types were syngeneic graft models (*n* = 77), followed by xenogeneic graft models (*n* = 31), chemically-induced (*n* = 4), virally induced (*n* = 1) and genetically engineered mouse (*n* = 1); no patient-derived xenograft models or carotid-/cardiac-injection metastasis models were identified. These can be further divided by species (Table 2). Again, we found an apparent near uniform consensus in that the most used species were rat and mouse, accounting for over 70% of all animal models (Table 3). While investigators seemed to favor immunocompetent rat strains, especially Fischer or Sprague–Dawley, there was marked variation in the utilization of murine strains. There was also considerable variation in the investigators’ choice of tumor implant; most investigators implanted cultured rat glioma cells (59 models) but there was considerable variation in the cell line used.

**Table 1:** Summary of animal model categories

Immunocompetent	Chemically-induced		Animals are exposed (often in utero) to carcinogens such as *N*-ethylnitrosurea or methylchloranthene to cause spontaneous generation of tumors in vivo. Tumors develop with delay and in an unpredictable fashion, with some animals developing several tumors while some may not develop any in the allocated time	[29, 49–55]
	Virally-induced		Animals are exposed to carcinogenic viruses such as Rous Sarcoma Virus to cause spontaneous generation of tumors	[56]
	Implanted/Injected (syngeneic)	From culture	Tumors induced in animals of the same species as the prospective host organism are taken and stored in cell culture prior to transplantation, e.g. F98, 9L, C6 or RG2 cell lines into rat; B16F10 into mouse, VX2 into rabbit	[36, 53–61]
		From tumor biopsy	Tumors induced in animals of the same species as the prospective host organism are taken from a tumor-bearing animal and implanted into another of the same species. The tumors may be spontaneous or may have been induced by other means, e.g. chemical	[62]
	Implanted/Injected (xenogeneic)	From culture	Tumors induced in animals of a different species as the prospective host organism are taken and stored in cell culture prior to transplantation, e.g. rat lines like RG2 and F98 in outbred cat	[50, 51, 63]
	Genetically-engineered animals		Animals are genetically engineered to overexpress oncogenes or lose critical tumor suppressor gene function meaning that tumors can be generated either spontaneously or by use of triggers	[13]
Immunocompromised	Implanted/injected (xenogeneic)	From culture	Tumor cells obtained from animals of a different species as the prospective host organism are propagated in cell culture prior to transplantation into an immunocompromised animals (i.e. mice and rats), e.g. human cell lines like U87, LX1 etc. or mice or rat cell lines such as C6	[28, 64]
		From tumor biopsy	Tumors obtained from animals of a different species as the prospective host organism are transplanted directly into an immunocompromised animal	Not represented in this review

**Table 2:** Number of animal models reported by species and species strain of tumor-induction method used

Tumor model Species–cell line/origin	No of reports	Tumor model Species–cell line/origin	No of reports
Rat—C6	22	Rat—RGl2.2	3
Rat—RG2	11	Rat—Induced	3
Rat—9L	11	Rat—Walker 256	2
Rat—F98	10	Rat—A15A5	2
Rat—RN6	2	Rat—Miscellaneous	8
Human—Miscellaneous	9	Mouse—B16F10	2
Human—U87	5	Mouse—Miscellaneous	8
Rabbit—Miscellaneous	1	Mouse—GEM	1
Rabbit—VX2	7	Mouse—Induced	1
Dog—Miscellaneous	1	Other—Induced	1
Unknown etiology	4		

All injected/implanted cell lines were implanted intracranially, no models used intracarotid or -cardiac injection

**Table 3:** Number of animal models reported by species and species strain of tumor-bearing animal used

Host species	Total (species)	Immune status	No of reports	Strain	No of reports
Rat	61	Competent	57	Fischer	30
				Sprague–Dawley	10
				Wistar	9
				BD-IX	6
				NOS	2
		Compromised	4	Nude	4
Mouse	26	Competent	12	C57 Bl/6 variants	7
				Knockout mice	2
				C3H	1
				VM/Dk	1
				NOS	1
		Compromised	14	Other nude	8
				BALB/c nude	6
Rabbit	8				
Macaque	1				
Dog	2				
Guinea pig	4				
Cat	12				

All injected/implanted cell lines were implanted intracranially, no models used intracarotid or -cardiac injection

The most common combination of tumor-bearing animal and method of tumor generation was a variant of a rat-derived tumor cell line implanted into immunocompetent rats, accounting for 53 of all animal models. For example, the single biggest subgroup, defined as using the same strain and same cell line were Fischer rats implanted with 9L rat glioma cells but this accounted for only 9 of the 61 individual studies using rats.

Finally, there was variation in the methodology used (Table 4) for assessing the extent of edema, and, where applicable, response to treatment. The most prevalent method was assessment by histopathology (70% of studies) with studies of BWC and extravasation reported in just over 40% of studies, symptomatology in 38%, imaging in 31%, electrolyte composition assays in 10% and permeability studies in 6% of animal models.

**Table 4:** Summary of peritumoral edema measures

Edema measure	Description
Histology	We included studies that used any type of histopathological technique. These included “simple” staining and light microscopy, immune-staining and light-microscopy as well as scanning- and transmission electromicrography techniques. Factors such as distance between cells can then be used as a measure of edema. Histopathological techniques require post-mortem tissue for analysis and therefore allow only “snapshot” sampling of edema. Histopathological techniques were frequently used concurrently with extravasation of immune-stainable substances not normally found in brain tissue, either endogenous (albumin) or exogenous (horseradish peroxidase) to provide some measure of “leakiness” of cerebral vasculature
Brain water content (BWC)	Brain water content studies predominantly use the difference between wet and dried weight of an aliquot of brain tissue to estimate the water content of the tissue. Tissue is harvested post-mortem, weighed and then dried according to protocol (usually in an oven) and weighed again afterwards. This method cannot distinguish between increased extracellular fluid, as seen in peritumoral vasogenic edema, and increased water content e.g. due to cytotoxic edema
Extravasation studies	Extravasation studies include any studies that endeavor to establish the concentration of a chemical that is not normally present in brain tissue but occurs either naturally in blood, or is injected. The most commonly used substances were dyes such as Evans blue, exogenous proteins like horseradish peroxidase and endogenous proteins like albumin. The presence or concentration of these can then be detected in tissue post-mortem by a variety of techniques such as immunostaining microscopy, Western blotting and spectroscopy. This technique is an indirect measure of blood–brain barrier “leakiness” rather than edema, but the former is often used by investigators as a proxy for the latter
Imaging	Imaging can be used to investigate animals in vivo for radiographic appearances suggestive of tumor and/or peritumoral edema. The most common imaging modality is MRI where one would expect areas of high T2/FLAIR and low T1 signal relative to surrounding grey/white matter around the tumor to correspond to edema. However, imaging interpretation can be complex, especially in tumors that form complex heterogeneous masses which makes interpretation of imaging more difficult. MRI scans are considerably more expensive than the other techniques outlined here
Symptomatology	Assessing symptomatology in animals can be done in a number of ways; the simplest is to use a measure of mortality e.g. the time from the tumor instigation until death. More in-depth techniques have been used in some studies, for instance, observing for unilateral weakness or pupillary mydriasis (signs of considerable localizing mass effect) or observing for behavioral change (failure to feed, failure to groom). This has the benefit of measuring what would likely constitute a primary outcome in any human trial (morbidity/mortality) and can be done cheaply and repeatedly over time. It should be noted, however, that these measures do not differentiate between tumor growth and isolated tumor edema
Electrolyte composition assay	Similar to BWC, an aliquot of brain tissue is homogenized and ashened before quantifying the electrolyte content for instance with liquid chromatography. This technique is occasionally seen reported together with BWC, with which it shares significant methodological overlap
Permeability studies	We included in this category studies that used infusion of substances such as radio-labelled small molecules like aminoisobutyric acid, to allow calculation of a permeability constant. This is a more formal quantification of the permeability of brain vasculature to that particular substance than can be obtained by simply injecting a bolus of a substance as with extravasation studies. These techniques require sacrificing the animal

Of the 24 articles containing MR images, 11 had no clear peritumoral edema (all rodent models), 4 showed a pattern of peritumoral halo all with edema volume less than tumor volume (3 rat glioma clone F98 tumor models, 1 rat 9L gliosarcoma model), 6 revealed infiltrative edema with volume less than tumor volume (4 rats, 1 mouse, 1 dog) and the remaining 3 (all cat models) showed infiltrative edema with greater volume than the tumor (Table 5). Eleven of the 24 studies where involved the assessment of an anti-edema agent.

**Table 5:** Table classifying extent of peritumoral edema as shown on the MR images provided in studies of animal brain tumor models

No	1^st^ Author (year)	Animal	Tumor model	MRI magnet strength (Tesla, T)	Images provided	Edema pattern
1	Yang et al. [39]*	Mouse (4–6-week-old BALB/c (*nu/nu*) mice)	Rat C6 glioma cells	0.5 T	T1 pre & post Gd, T2	No clear edema
2	Yamamoto et al. [38]	Rat (Fischer)	Rat 9L glioma/gliosarcoma cells	2.4 T	T1 post-ATN-10	No clear edema
3	Whelan et al. [43]	Dog	Canine gliosarcoma cells	0.5 T	T1 pre & post Gd, T2	Infiltrative, less than tumor volume
4	Tjujajev et al. [15]*	Rat (Fischer)	Rat RG2 gliomas	4.7 T	T1 post Gd, proton density	Infiltrative, less than tumor volume
5	Tjuvajev et al. [62]	Rat (Fischer)	Rat RG2 gliomas	4.7 T	T1 post Gd	No clear edema
6	Thompson et al. [21]	Rat (nude)	Human Small cell lung carcinoma (SCLC) LX1 or A2058 melanoma cells	11.75 T	T1 pre & post Gd DCE-MRI	No clear edema
7	Takahashi et al. [37]	Rat (Wistar)	Rat C6 glioma cells	Not specified	T1 pre & post Gd, T2	Infiltrative, less than tumor volume
8	Shevtsov et al. [22]	Rat (Wistar)	Rat C6 glioma cells	Not specified	T1, T2 and DWI with ADC maps	No clear edema
9	Sehm et al. [34]*	Rat (Fischer)	Rat glioma clone F98	3 T	T1 post Gd & T2	Peritumoral halo, less than tumor volume
10	Pitter et al. [13]*	Mouse (Ntv-a/ink4a-arf-/- and Gli-luc;Ntv-a;Ink4a-Arf-/- mice)	RCAS-PDGFB-HA-transfected DF-1 cell suspension	9.4 T	T1 post Gd & T2	Infiltrative, less than tumor volume
11	Mazurchuk et al. [36]	Rat (Fisher)	Rat 9L glioma/gliosarcoma	1.5 T	T1 & T2	Peritumoral halo, less than tumor volume
12	Li et al. [35]*	Rat (Wistar)	Rat C6 glioma cells	3 T	T2	No clear edema
13	Kamoun et al. [25]*	Mouse (Nude)	Human U87 or U118 or Rat CNS1 tumors	9.4 T	T2	No clear edema
14	Ito et al. [60]	Rat (Wistar)	Rat C6 glioma cells	1.5 T	T1 post-Gd	No clear edema
15	Hossmann et al. [23]	Cat	Rat glioma clone F98	Not specified	T1 pre & post Gd, T2	Infiltrative, greater than tumor volume
16	Hoehn-Berlage et al. [41]	Rat (Fischer)	Rat glioma clone F98	4.7 T	IR snapshot FLASH images pre and post MnTPPS	Infiltrative, less than tumor volume
17	Hoehn-Berlage et al. [52]	Cat	Rat glioma clone F98	4.7 T	T2	Infiltrative, greater than tumor volume
18	Hoehn-Berlage et al. [32]	Rat (Fischer)	Rat glioma (F98), schwannoma (RN6), or neuroblastoma (E367)	4.7 T	T2 & T1 post Gd	Infiltrative, less than tumor volume
19	Engelhorn et al. [31]	Rat (Fischer)	Rat glioma clone F98	1.5 T	CISS, T2, T1 post Gd	Peritumoral halo, less than tumor volume
20	Eis et al. [59]	Rat (Fischer)	Rat F98 glioma, RN6 schwannoma and E367 neuroblastoma	4.7 T	T1, T2, PD, ADC maps	No clear edema
21	Chae et al. [28]*	Mouse (Nude)	Human U87 glioma cells	9.4 T	T2	No clear edema
22	Bulnes et al. [29]*	Rat (Sprague–Dawley)	Ethylnitrosourea (ENU) administration	Not specified	T1 post Gd & T2	No clear edema
23	Bockhorst et al. [30]	Rat (Fischer)	Rat glioma clone F98	4.7 T	T1 pre & post Gd, T2	Peritumoral halo, less than tumor volume
24	Bayens-Simmonds et al.[42]	Cat	9L glioma/gliosarcoma	2.35 T	Varying echo pulse	Infiltrative, greater than tumor volume

*MnTPPS*  Tumor-enhancing Contrast agent manganese(III) tetraphenylporphine sulfonate. *Gd* Gadolinium, intravenous contrast agent

ATN-10 (Manganese-metalloporphyrin)

*Studies involved in the assessment of an anti-edema agent

## Discussion

We here report the findings from a systematic search and review of the literature on animal brain tumor models in which brain edema was reported. Most of the studies reviewed used indirect measures of cerebral edema (BWC, pathology, blood–brain barrier permeability) and these may not equate to the edema seen in patients with high grade tumors. Even in those studies where radiology was used, few of the animal models showed peritumoral brain edema in keeping with that seen in patients—and in the models which did show impressive edema, most notably in an immunocompetent feline model with an implanted xenograft tumor, there would be concern that the mechanism of edema formation is different to that seen in glioma. Overall, we found no evidence for a definitive animal model to use when assessing changes in peritumoral brain edema following treatment. The reports that have been published show considerable heterogeneity in design; this likely reflects peritumoral edema having been of secondary concern in designing brain tumor animal models.

Our results demonstrate that there is considerable heterogeneity in the combinations of tumor-bearing animal and tumor-induction method combinations and the measures used to assess the extent of edema. Edema measures in the literature presented here can be characterized as measuring BWC, measuring blood vessel leakiness, measuring symptoms or detecting radiological signs of edema (Table 4). There was often a disconnect between the results of these measures. For instance, Kamoun et al. [25] used BWC, imaging and clinical outcomes taken together in an immunosuppressed mouse model implanted with cultured human-derived glioma-derived cell lines. In their study, there was a significant increase in BWC in the ipsilateral cerebral hemisphere of tumor-bearing animals compared with tumor-free controls and this reduced following treatment with cediranib. Cediranib, which inhibits vascular endothelial growth factor (VEGF) receptor tyrosine kinase, limiting the growth of new blood vessels, also significantly delayed mortality in this model. However, the MRI images presented appeared not to show convincing T2 signal attributable to edema. This suggests that in this model, BWC changes occur without radiological evidence of peritumoral brain edema. This disconnect is important because most animal studies use histopathology and BWC to estimate peritumoral brain edema with imaging only occasionally being used. By contrast, in clinical practice, the reverse is true: clinical signs and radiology are routinely used to inform treatment decisions.

The radiological appearances of edema in the animal models were also very different compared to that seen in humans. On review of the imaging shown in the reports selected in this review, none of the animal tumor models showed a clear resemblance to those of humans with high grade brain tumors. The majority showed no peritumoral edema although some did show a halo pattern of edema. The halo gives the impression of a capsule or boundary within which the edema is confined. In contrast, in patients with GBM and metastases there is often widespread edema which migrates along the white matter tracts. In human clinical trials there are no standardized criteria for measuring/recording this cerebral edema—although there are standardized methods for recording radiological response to treatment (e.g. RANO [26]). Carlson et al. [27] devised a grading system for peritumoral brain edema:—grade 0 being no edema; grade 1 showing edema up to 2 cm from the tumor margin and grade 3 having edema extending more than 2 cm from the tumor margin. These authors noted that in GBM patients, 23% were grade 0, 23% were grade 1 and 54% showed grade 2, edema i.e. over 75% of patients had significant peritumoral brain edema.

In the animal models reviewed here, the extent of peritumoral edema appeared to be model dependent, with immunocompromised animals, perhaps not surprisingly, showing essentially no T2 signal attributable to peritumoral brain edema [25, 28]. Genetically-engineered mouse models and ENU-induced tumors in rats also showed no MRI evidence of edema [13, 29]. In immunocompetent rats implanted with rat-derived tumors only relatively minor edema is seen surrounding the tumor, often with a peritumoral ‘halo’, with the ratio of tumor volume to edema volume reported as between 50 and 150% [30–34]. In other reports, no or almost no T2 signal attributable to peritumoral edema can be seen on images in the manuscript [35–39]. This is different to human high grade tumors where it is common to see edema propagating through white matter, reaching large volumes and sometimes even crossing the midline into the macroscopically tumor-free hemisphere [40]. Most of the models discussed above have demonstrated edema using measures such as histopathological assessment or BWC, or by studying blood vessel permeability. Given that the extent of peritumoral edema in humans is often vastly greater radiologically, it is questionable how well these other measures would predict treatment response in humans.

We have found only one group of animal model that appears to show peritumoral edema similar to that seen in humans, namely cats injected with one of several rat glioma cell lines, e.g. F98 [41], RG2, 9L or C6. Studies have shown that the edema occurs preferentially in white matter and has increased albumin content, confirming the vasogenic origin of the fluid in these models [42].

It is not clear what drives these differences in peritumoral brain edema between the different animal models and between pre-clinical and clinical appearances. We can propose several possible explanations. Firstly, small rodents have a comparatively limited amount of white matter in the brain compared to larger animals. As radiologically demonstrable edema in humans has a predilection for white matter tracts, this may affect the limited amount of edema visible in rodent models. This may be supported by reports of spontaneously occurring canine gliomas which show considerable white matter tract edema [43] and by the edema seen in feline models. Furthermore, there may be less apparent intrinsic differences in the preponderance of different species’ organs for developing tumors [44, 45]—given that spontaneous brain tumors in rodents are very rare but do occur in larger animals, e.g. dogs [43, 46], there may be hitherto undescribed differences in the brains of small rodents and other animals that lead to different behavior of brain tumors, including edema. It must also be noted that the most common implantation site in rodents is the dorsal striatum which is different from where we see tumors in patients and may have an effect of the initiation and expansion of edema.

Secondly, there are considerable differences in immune status between the different models. Given the importance of inflammatory mediators in generating vasogenic edema, this may account for at least some of the variability in peritumoral brain edema. In the model where a rat or mouse-derived xenograft is implanted into immunocompetent cats, considerable edema is seen. Interestingly, while there is good evidence that the rat brain is an immune privileged site [47–49] and does not tend to generate much of an immune response to injected tissue unless the tumor material contacts non-brain parenchyma (such as in infiltration into the cerebral ventricles, or concurrent subdermal and intracerebral injection). By comparison, cats injected with rat tumor lines show features of immunological rejection [50] with leuko- and lymphocytic infiltration, which strongly implies the cat immune system behaves differently to that of rats. One would expect a xenograft to generate a near-maximal immunological reaction; this immune response and/or rejection may contribute to loss of integrity of the blood brain barrier and an edematous reaction resembling that seen in humans with gliomata in effect but produced by a mechanism that is not proven to be similar to human peritumoral brain edema and may be more akin to the edema seen in patients with cerebral abscess. Conversely, immunocompetent rats implanted with rat glioma cell lines had much less edema, and immunocompromised rats generated no edema. The clinical course in these experiments mirrors the radiological findings, with the immunocompetent cats bearing xenografts having a relatively fulminant course with neurological decline in the initial 2 weeks followed by either death or recovery and eventual regression of the tumor [51], which is compatible with immune clearance of the implanted foreign material and does not reflect the experience of patients with high grade tumors.

We note that the studies included in this systematic search do not encompass all of the animal model literature: we present a disproportionate number of studies that model gliomata compared with models of metastases, and a notable paucity or absence of certain models. For instance, we present only one genetically-engineered mouse model, and our search did not return any porcine models, despite their existence and the presence of edema on scans [65]. Given our robust and inclusive search strategy, we believe this lends further credence to the hypothesis that the study of edema in animal model research has been limited.

In summary, differences in the edema generated appear to be partly driven by factors relating to immunogenicity of the tumor/tumor-bearing animal combination, but also the macrostructure of the brain of the tumor-bearing animal. Further, the implanted tumors behave somewhat unpredictably, with considerable between-subjects differences reported in the growth rate of the tumor and the degree of associated brain edema [42].

To conclude, we have shown that there are considerable limitations to the use of the brain tumor animal models presented herein to study tumor-induced brain edema and hence to study potential alternatives to steroids treatment.

We postulate that for an animal model of peritumoral edema to be considered valid, it should fulfill the following criteria:

(i) The tumor must produce an immune response that credibly mimics that of human brain to human tumor, i.e. it must not be driven by a xenogeneic immune reaction.
(ii) It must produce a relevant volume of radiologically detectable edema, with manifestations that temporarily improve or resolve with steroid treatment with corresponding changes in other measures of edema such as BWC, symptomatology, extravasation studies and so forth.
(iii) The model should be affordable and compatible with the highest standards of animal welfare.

Regrettably, none of the existing models of peritumoral brain edema reviewed here satisfy these fundamental requirements. However, there is opportunity here: there are tumor animal models have not been formally assessed as models of brain tumor edema yet, but could prove to be useful. We have highlighted two key areas of interest: immunocompetent models, and large animal models. Meanwhile, unless a relevant peritumoral brain edema animal model can be characterised, it is likely that potential alternatives to steroids will need to be trialed directly on patients. We would also argue that the neuro-oncology community should agree on standard outcome measures for recording peritumoral brain edema in both animal models and in patients on clinical trials.

## Appendix A-Search strategies

### Medline

- 1 Medline ("brain edema").ti,ab 5880
- 2 Medline "BRAIN EDEMA"/ 13,665
- 3 Medline ("brain hematoma").ti,ab 35
- 4 Medline "HEMATOMA, EPIDURAL, CRANIAL"/ 3331
- 5 Medline ("brain edema").ti,ab 857
- 6 Medline ("brain swelling").ti,ab 1469
- 7 Medline ("cerebral edema").ti,ab 4674
- 8 Medline ("cerebral edema").ti,ab 1160
- 9 Medline ("cerebral swelling").ti,ab 258
- 10 Medline ("cytotoxic brain edema").ti,ab 113
- 11 Medline ("intracranial edema").ti,ab 14
- 12 Medline ("vasogenic brain edema").ti,ab 252
- 13 Medline ("vasogenic cerebral edema").ti,ab 53
- 14 Medline (1 OR 2 OR 3 OR 4 OR 5 OR 6 OR 7 OR 8 OR 9 OR 10 OR 11 OR 12 OR 13) 23,492
- 15 Medline (astrocytoma).ti,ab 10,355
- 16 Medline ASTROCYTOMA/ 13,983
- 17 Medline (astroglioma).ti,ab 195
- 18 Medline ("brain cancer").ti,ab 2419
- 19 Medline "BRAIN NEOPLASMS"/ 100,154
- 20 Medline (brain AND (tumor* OR tumor*)).ti,ab 71,923
- 21 Medline ((brain AND (tumor* OR tumor*)) AND metastasis).ti,ab 4989
- 22 Medline ("brain neoplasm*").ti,ab 764
- 23 Medline ("brain neoplasm*" AND benign).ti,ab 38
- 24 Medline ("brain stem" AND (tumor* OR tumor*)).ti,ab 1842
- 25 Medline "BRAIN STEM NEOPLASMS"/ 1453
- 26 Medline ((brain AND (tumor* OR tumor*)) AND malignan*).ti,ab 14,988
- 27 Medline ((brain AND (tumor* OR tumor*)) AND primary).ti,ab 14,143
- 28 Medline ((brain AND (tumor* OR tumor*)) AND recurrent).ti,ab 2936
- 29 Medline ("cerebellar neoplasm*").ti,ab 46
- 30 Medline "CEREBELLAR NEOPLASMS"/ 8589
- 31 Medline (cerebellar AND (tumor* OR tumor*)).ti,ab 4313
- 32 Medline (cerebellum AND (tumor* OR tumor*)).ti,ab 2449
- 33 Medline ("cerebral astrocytoma").ti,ab 114
- 34 Medline ("cerebral ventricle" AND (tumor* OR tumor*)).ti,ab 54
- 35 Medline "CEREBRAL VENTRICLE NEOPLASMS"/ 3390
- 36 Medline ("cerebral ventricle" AND neoplasm*).ti,ab 2
- 37 Medline (cerebroventricular AND neoplasm*).ti,ab 0
- 39 Medline ("cerebellopontine" AND (tumor* OR tumor*)).ti,ab 2045
- 40 Medline ("choroid plexus neoplasm*").ti,ab 37
- 41 Medline "CHOROID PLEXUS NEOPLASMS"/ 597
- 42 Medline ("choroid plexus" AND (tumor* OR tumor*)).ti,ab 1396.
- 43 Medline (ependymoma).ti,ab 3316
- 44 Medline EPENDYMOMA/ 4715
- 45 Medline (ependymoblastoma).ti,ab 246
- 46 Medline "NEOPLASMS, NEUROEPITHELIAL"/ 807
- 47 Medline ("fibrillary astrocytoma").ti,ab 167
- 48 Medline (ganglioglioma).ti,ab 947
- 49 Medline GANGLIOGLIOMA/ 828
- 50 Medline ("gemistocytic astrocytoma").ti,ab 68
- 51 Medline (glioma).ti,ab 36,642
- 52 Medline GLIOMA/ 34,564
- 53 Medline (glioma AND astrocytic).ti,ab 785
- 54 Medline (glioma AND mixed).ti,ab 602
- 55 Medline (glioma AND subependymal).ti,ab 58
- 56 Medline (glioblastoma).ti,ab 26,720
- 57 Medline GLIOBLASTOMA/ 20,942
- 58 Medline ("glial cell" AND (tumor* OR tumor*)).ti,ab 699
- 59 Medline ("hypothalamic cancer").ti,ab 0
- 60 Medline ("hypothalamic neoplasm*").ti,ab 6
- 61 Medline ("hypothalamic teratoma").ti,ab 0
- 62 Medline "HYPOTHALAMIC NEOPLASMS"/ 712
- 63 Medline (hypothalamus AND (tumor* OR tumor*)).ti,ab 1650
- 64 Medline (hypophysis AND (tumor* OR tumor*)).ti,ab 310
- 65 Medline ("infratentorial cancer").ti,ab 0
- 66 Medline "INFRATENTORIAL NEOPLASMS"/ 781
- 67 Medline ("infratentorial neoplasm*").ti,ab 4
- 68 Medline (infratentorial AND (tumor* OR tumor*)).ti,ab 1001
- 69 Medline ("intracranial astrocytoma").ti,ab 12
- 70 Medline ("intracranial neoplasm*").ti,ab 1053
- 71 Medline (glioma AND malignan*).ti,ab 9955
- 72 Medline ("medullary neoplasm*").ti,ab 19
- 73 Medline (medullary AND (tumor* OR tumor*)).ti,ab 6216
- 74 Medline "NEOPLASMS, DUCTAL, LOBULAR, AND MEDULLARY"/ 70
- 75 Medline (medulloepithelioma).ti,ab 316
- 76 Medline MEDULLOBLASTOMA/ 639
- 77 Medline ("mesencephalic neoplasm*").ti,ab 0
- 78 Medline (midbrain AND (tumor* OR tumor*)).ti,ab 538
- 79 Medline (midbrain AND neoplasm*).ti,ab 49
- 80 Medline ("myxopapillary ependymoma").ti,ab 309
- 81 Medline "GLIOMA, SUBEPENDYMAL"/ 166
- 82 Medline (neurocytoma).ti,ab 596
- 83 Medline NEUROCYTOMA/ 560
- 84 Medline (neuroectodermal AND (tumor* OR tumor*)).ti,ab 5157
- 85 Medline exp "NEUROECTODERMAL TUMORS"/ 265,949
- 86 Medline (neurohypophysial AND neoplasm*).ti,ab 4
- 87 Medline (oligoastrocytoma).ti,ab 478
- 88 Medline (oligoastrocytic AND (tumor* OR tumor*)).ti,ab 37
- 89 Medline (oligodendroglioma).ti,ab 2318
- 90 Medline OLIGODENDROGLIOMA/ 3409
- 91 Medline (oligodendrocytosis).ti,ab 5
- 92 Medline (parenchymal AND (tumor* OR tumor*)).ti,ab 3837
- 93 Medline PINEALOMA/ 1782
- 94 Medline ("pilocytic astrocytoma").ti,ab 1211
- 95 Medline ("pineal gland" AND (tumor* OR tumor*)).ti,ab 580
- 96 Medline (pineoblastoma).ti,ab 295
- 97 Medline (pinealoma).ti,ab 251
- 98 Medline (pineocytoma).ti,ab 187
- 99 Medline (PNET).ti,ab 2115
- 100 Medline ("primitive neuroectodermal" AND (tumor* OR tumor*)).ti,ab 3212
- 101 Medline ("pons angle" AND (tumor* OR tumor*)).ti,ab 2
- 102 Medline ("pontine neoplasm").ti,ab 2
- 103 Medline (pontine AND (tumor* OR tumor*)).ti,ab 970
- 104 Medline (pontine AND glioma).ti,ab 505
- 105 Medline ("posterior fossa" AND neoplasm).ti,ab 110
- 106 Medline ("posterior fossa" AND (tumor* OR tumor*)).ti,ab 2887
- 107 Medline (spongioblastoma).ti,ab 109
- 108 Medline "NEUROECTODERMAL TUMORS, PRIMITIVE"/ 1661
- 109 Medline (subependymoma).ti,ab 265
- 110 Medline ((subtentorial AND tumor*) AND tumor*).ti,ab 2
- 111 Medline ("supratentorial neoplasm*").ti,ab 28
- 112 Medline "SUPRATENTORIAL NEOPLASMS"/ 2001
- 113 Medline (tentorial AND meningioma).ti,ab 176
- 114 Medline (cerebri AND (tumor* OR tumor*)).ti,ab 484
- 115 Medline (ventrical AND (tumor* OR tumor*)).ti,ab 8
- 116 Medline (ventricular AND (tumor* OR tumor*)).ti,ab 5805
- 117 Medline (15 OR 16 OR 17 OR 18 OR 19 OR 20 OR 21 OR 22 OR 23 OR 24 OR 25 OR 26 OR 27 OR 28 OR 29 OR 30) 128,452
- 118 Medline (31 OR 32 OR 33 OR 34 OR 35 OR 36 OR 39 OR 40 OR 41 OR 42 OR 43 OR 44 OR 45 OR 46 OR 47 OR 48 OR 49 OR 50 OR 51 OR 52 OR 53 OR 54 OR 55 OR 56 OR 57 OR 58 OR 60 OR 62 OR 63 OR 64) 90,403
- 119 Medline (66 OR 67 OR 68 OR 69 OR 70 OR 71 OR 72 OR 73 OR 74 OR 75 OR 76 OR 78 OR 79 OR 80 OR 81 OR 82 OR 83 OR 84 OR 85 OR 86 OR 87 OR 88 OR 89 OR 90) 276,190
- 120 Medline (91 OR 92 OR 93 OR 94 OR 95 OR 96 OR 97 OR 98 OR 99 OR 100 OR 101 OR 102 OR 103 OR 104 OR 105 OR 106 OR 107 OR 108 OR 109 OR 110 OR 111 OR 112 OR 113 OR 114 OR 115 OR 116) 23,358
- 121 Medline (14 AND 117) 1867
- 122 Medline (14 AND 118) 864
- 123 Medline (14 AND 119) 944
- 124 Medline (14 AND 120) 195
- 125 Medline (animal* OR "animal health" OR "animal population" OR "animal research" OR "animal study" OR "animal studies" OR "laboratory animal" OR primate OR rabbit OR rodent OR rat).ti,ab 1,865,196
- 126 Medline ANIMALS/ OR "ANIMAL POPULATION GROUPS"/ 6,167,987
- 127 Medline "MODELS, ANIMAL"/ OR "ANIMAL EXPERIMENTATION"/ 41,734
- 128 Medline (125 OR 126 OR 127) 6,410,366
- 129 Medline (121 AND 128) 300
- 130 Medline (122 AND 128) 195
- 131 Medline (123 AND 128) 203
- 132 Medline (124 AND 128) 12
- 133 Medline 129 [Animals] 284
- 134 Medline 130 [Animals] 185
- 135 Medline 131 [Animals] 193
- 136 Medline 132 [Animals] 11

### PubMed

- 1 PubMed ("brain edema").ti,ab 16,319
- 2 PubMed ("brain hematoma").ti,ab 55
- 3 PubMed ("brain edema").ti,ab 864
- 4 PubMed ("brain swelling").ti,ab 1484
- 5 PubMed ("cerebral edema").ti,ab 4773
- 6 PubMed ("cerebral edema").ti,ab 1172
- 7 PubMed ("cerebral swelling").ti,ab 260
- 8 PubMed ("cytotoxic brain edema").ti,ab 114
- 9 PubMed ("intracranial edema").ti,ab 15
- 10 PubMed ("vasogenic brain edema").ti,ab 253
- 11 PubMed ("vasogenic cerebral edema").ti,ab 54
- 12 PubMed (1 OR 2 OR 3 OR 4 OR 5 OR 6 OR 7 OR 8 OR 9 OR 10 OR 11) 20,554
- 13 PubMed (astrocytoma).ti,ab 35,937
- 14 PubMed (astroglioma).ti,ab 36,037
- 15 PubMed ("brain cancer").ti,ab 2644
- 16 PubMed (brain AND (tumor* OR tumor*)).ti,ab 7655
- 17 PubMed ((brain AND (tumor* OR tumor*)) AND metastasis).ti,ab 8827
- 18 PubMed ("brain neoplasm*").ti,ab 289
- 19 PubMed ("brain neoplasm*" AND benign).ti,ab 7
- 20 PubMed ("brain stem" AND (tumor* OR tumor*)).ti,ab 411
- 21 PubMed ((brain AND (tumor* OR tumor*)) AND malignan*).ti,ab 20,587
- 22 PubMed ((brain AND (tumor* OR tumor*)) AND primary).ti,ab 20,651
- 23 PubMed ((brain AND (tumor* OR tumor*)) AND recurrent).ti,ab 4955
- 24 PubMed ("cerebellar neoplasm*").ti,ab 23
- 25 PubMed (cerebellar AND (tumor* OR tumor*)).ti,ab 948
- 26 PubMed (cerebellum AND (tumor* OR tumor*)).ti,ab 464
- 27 PubMed ("cerebral astrocytoma").ti,ab 121
- 28 PubMed ("cerebral ventricle" AND (tumor* OR tumor*)).ti,ab 234
- 29 PubMed ("cerebral ventricle" AND neoplasm*).ti,ab 3420
- 30 PubMed (cerebroventricular AND neoplasm*).ti,ab 2
- 31 PubMed ("choroid plexus neoplasm*").ti,ab 5
- 32 PubMed ("choroid plexus" AND (tumor* OR tumor*)).ti,ab 119
- 33 PubMed (ependymoma).ti,ab 6103
- 34 PubMed (ependymoblastoma).ti,ab 37,355
- 35 PubMed ("fibrillary astrocytoma").ti,ab 166
- 36 PubMed (ganglioglioma).ti,ab 1296
- 37 PubMed ("gemistocytic astrocytoma").ti,ab 70
- 38 PubMed (glioma).ti,ab 84,055
- 39 PubMed (glioma AND astrocytic).ti,ab 2494
- 40 PubMed (glioma AND mixed).ti,ab 1474
- 41 PubMed (glioma AND subependymal).ti,ab 622
- 42 PubMed (glioblastoma).ti,ab 33,206
- 43 PubMed ("glial cell" AND (tumor* OR tumor*)).ti,ab 40
- 44 PubMed ("hypothalamic cancer").ti,ab 0
- 45 PubMed ("hypothalamic neoplasm*").ti,ab 2
- 46 PubMed ("hypothalamic teratoma").ti,ab 0
- 47 PubMed (hypothalamus AND (tumor* OR tumor*)).ti,ab 517
- 48 PubMed (hypophysis AND (tumor* OR tumor*)).ti,ab 598
- 49 PubMed ("infratentorial cancer").ti,ab 0
- 50 PubMed ("infratentorial neoplasm*").ti,ab 2
- 51 PubMed (infratentorial AND (tumor* OR tumor*)).ti,ab 101
- 52 PubMed ("intracranial astrocytoma").ti,ab 12
- 53 PubMed ("intracranial neoplasm*").ti,ab 295
- 54 PubMed (glioma AND malignan*).ti,ab 18,717
- 55 PubMed ("medullary neoplasm*").ti,ab 6
- 56 PubMed (medullary AND (tumor* OR tumor*)).ti,ab 835
- 57 PubMed ("mesencephalic neoplasm*").ti,ab 0
- 58 PubMed (midbrain AND (tumor* OR tumor*)).ti,ab 89
- 59 PubMed (midbrain AND neoplasm*).ti,ab 1325
- 60 PubMed ("myxopapillary ependymoma").ti,ab 314
- 61 PubMed (neurocytoma).ti,ab 745
- 62 PubMed (neuroectodermal AND (tumor* OR tumor*)).ti,ab 419
- 63 PubMed (neurohypophysial AND neoplasm*).ti,ab 31
- 64 PubMed (oligoastrocytoma).ti,ab 4711
- 65 PubMed (oligoastrocytic AND (tumor* OR tumor*)).ti,ab 2
- 66 PubMed (oligodendrocytosis).ti,ab 6
- 67 PubMed (parenchymal AND (tumor* OR tumor*)).ti,ab 264
- 68 PubMed ("pilocytic astrocytoma").ti,ab 1265
- 69 PubMed ("pineal gland" AND (tumor* OR tumor*)).ti,ab 132
- 70 PubMed (pineoblastoma).ti,ab 1943
- 71 PubMed (pinealoma).ti,ab 1833
- 72 PubMed (pineocytoma).ti,ab 1879
- 73 PubMed (PNET).ti,ab 38,072
- 74 PubMed ("primitive neuroectodermal" AND (tumor* OR tumor*)).ti,ab 152
- 75 PubMed ("pons angle" AND (tumor* OR tumor*)).ti,ab 14
- 76 PubMed ("pontine neoplasm").ti,ab 2
- 77 PubMed (pontine AND (tumor* OR tumor*)).ti,ab 223
- 78 PubMed (pontine AND glioma).ti,ab 1058.
- 79 PubMed ("posterior fossa" AND neoplasm).ti,ab 3846.
- 80 PubMed ("posterior fossa" AND (tumor* OR tumor*)).ti,ab 244
- 81 PubMed (spongioblastoma).ti,ab 37,261
- 82 PubMed (subependymoma).ti,ab 483
- 83 PubMed ((subtentorial AND tumor*) AND tumor*).ti,ab 1
- 84 PubMed ("supratentorial neoplasm*").ti,ab 7
- 85 PubMed (tentorial AND meningioma).ti,ab 260
- 86 PubMed (cerebri AND (tumor* OR tumor*)).ti,ab 99
- 87 PubMed (ventrical AND (tumor* OR tumor*)).ti,ab 3
- 88 PubMed (ventricular AND (tumor* OR tumor*)).ti,ab 762
- 89 PubMed (69 OR 70 OR 71 OR 72 OR 73 OR 74 OR 75 OR 76 OR 77 OR 78 OR 79 OR 80 OR 81 OR 82 OR 83 OR 84 OR 85 OR 86 OR 87 OR 88) 45,658
- 90 PubMed (49 OR 50 OR 51 OR 52 OR 53 OR 54 OR 55 OR 56 OR 57 OR 58 OR 59 OR 60 OR 61 OR 62 OR 63 OR 64 OR 65 OR 66 OR 67 OR 68) 27,601
- 91 PubMed (29 OR 30 OR 31 OR 32 OR 33 OR 34 OR 35 OR 36 OR 37 OR 38 OR 39 OR 40 OR 41 OR 42 OR 43 OR 44 OR 45 OR 46 OR 47 OR 48) 122,455
- 92 PubMed (13 OR 14 OR 15 OR 16 OR 17 OR 18 OR 19 OR 20 OR 21 OR 22 OR 23 OR 24 OR 25 OR 26 OR 27 OR 28) 75,219
- 93 PubMed (animal* OR "animal health" OR "animal population" OR "animal research" OR "animal study" OR "animal studies" OR "laboratory animal" OR primate OR rabbit OR rodent OR rat).ti,ab 21,587,741
- 94 PubMed (12 AND 92 AND 93) 867
- 95 PubMed (12 AND 91 AND 93) 832
- 96 PubMed (12 AND 90 AND 93) 272
- 97 PubMed (12 AND 89 AND 93) 102
- 98 PubMed (human).ti,ab 17,558,986
- 99 PubMed 94 not 98 76
- 100 PubMed 95 not 98 121
- 101 PubMed 96 not 98 18
- 102 PubMed 97 not 98 8

### CINAHL

- 1 CINAHL ("brain edema").ti,ab 332
- 2 CINAHL "CEREBRAL EDEMA"/ 920
- 3 CINAHL ("brain hematoma").ti,ab 1
- 4 CINAHL ("brain swelling").ti,ab 94
- 5 CINAHL ("cerebral edema").ti,ab 406
- 6 CINAHL ("cerebral edema").ti,ab 84
- 7 CINAHL ("cerebral swelling").ti,ab 14
- 8 CINAHL ("cytotoxic brain edema").ti,ab 4
- 9 CINAHL ("intracranial edema").ti,ab 1
- 10 CINAHL ("vasogenic brain edema").ti,ab 4
- 11 CINAHL ("vasogenic cerebral edema").ti,ab 4
- 12 CINAHL (1 OR 2 OR 3 OR 4 OR 5 OR 6 OR 7 OR 8 OR 9 OR 10 OR 11) 1367
- 13 CINAHL (astrocytoma).ti,ab 333
- 14 CINAHL (astroglioma).ti,ab 1
- 15 CINAHL ("brain cancer").ti,ab 263
- CINAHL "BRAIN NEOPLASMS"/ 4757
- 17 CINAHL (brain AND (tumor* OR tumor*)).ti,ab 3251
- 18 CINAHL ((brain AND (tumor* OR tumor*)) AND metastasis).ti,ab 393
- 19 CINAHL ("brain neoplasm*").ti,ab 39
- 20 CINAHL ("brain neoplasm*" AND benign).ti,ab 1
- 21 CINAHL ("brain stem" AND (tumor* OR tumor*)).ti,ab 29
- 22 CINAHL ((brain AND (tumor* OR tumor*)) AND malignan*).ti,ab 534
- 23 CINAHL ((brain AND (tumor* OR tumor*)) AND primary).ti,ab 723
- 24 CINAHL ((brain AND (tumor* OR tumor*)) AND recurrent).ti,ab 139
- 25 CINAHL ("cerebellar neoplasm*").ti,ab 1
- 26 CINAHL (cerebellar AND (tumor* OR tumor*)).ti,ab 122
- 27 CINAHL (cerebellum AND (tumor* OR tumor*)).ti,ab 61
- 28 CINAHL ("cerebral astrocytoma").ti,ab 1
- 29 CINAHL ("cerebral ventricle" AND (tumor* OR tumor*)).ti,ab 1
- 30 CINAHL ("cerebral ventricle" AND neoplasm*).ti,ab 0
- 31 CINAHL (cerebroventricular AND neoplasm*).ti,ab 0
- 32 CINAHL (cerebellopontine AND (tumor* OR tumor*)).ti,ab 68
- 33 CINAHL ("choroid plexus neoplasm*").ti,ab 0
- 34 CINAHL ("choroid plexus" AND (tumor* OR tumor*)).ti,ab 18
- 35 CINAHL (ependymoma).ti,ab 182
- 36 CINAHL (ependymoblastoma).ti,ab 0
- 37 CINAHL ("fibrillary astrocytoma").ti,ab 2
- 38 CINAHL (ganglioglioma).ti,ab 21
- 39 CINAHL (glioma).ti,ab 1326
- 40 CINAHL GLIOMA/ 2607
- 41 CINAHL (glioma AND astrocytic).ti,ab 20
- 42 CINAHL (glioma AND mixed).ti,ab 28
- 43 CINAHL (glioma AND subependymal).ti,ab 1
- 44 CINAHL (glioblastoma).ti,ab 1097
- 45 CINAHL ("glial cell" AND (tumor* OR tumor*)).ti,ab 22
- 46 CINAHL ("hypothalamic cancer").ti,ab 0
- 47 CINAHL ("hypothalamic neoplasm*").ti,ab 0
- 48 CINAHL ("hypothalamic teratoma").ti,ab 0
- 49 CINAHL (hypophysis AND (tumor* OR tumor)).ti,ab 1
- 50 CINAHL ("infratentorial cancer").ti,ab 0
- 51 CINAHL "INFRATENTORIAL NEOPLASMS"/ 73
- 52 CINAHL "HYPOTHALAMIC NEOPLASMS"/ 9
- 53 CINAHL ("infratentorial neoplasm*").ti,ab 1
- 54 CINAHL (infratentorial AND (tumor* OR tumor*)).ti,ab 17
- 55 CINAHL ("intracranial astrocytoma").ti,ab 1
- 56 CINAHL ("intracranial neoplasm*").ti,ab 37
- 57 CINAHL (glioma AND malignan*).ti,ab 358
- 58 CINAHL ("medullary neoplasm*").ti,ab 0
- 59 CINAHL (medullary AND (tumor* OR tumor*)).ti,ab 134
- 60 CINAHL "NEOPLASMS, DUCTAL, LOBULAR, AND MEDULLARY"/ 324
- 61 CINAHL (medulloepithelioma).ti,ab 3
- 62 CINAHL "NEUROECTODERMAL TUMORS, PRIMITIVE"/ 80
- 63 CINAHL (medulloblastoma).ti,ab 284
- 64 CINAHL ("mesencephalic neoplasm*").ti,ab 0
- 65 CINAHL (midbrain AND (tumor* OR tumor*)).ti,ab 16
- 66 CINAHL (midbrain AND neoplasm*).ti,ab 2
- 67 CINAHL ("myxopapillary ependymoma").ti,ab 16
- 68 CINAHL (neurocytoma).ti,ab 29
- 69 CINAHL (neuroectodermal AND (tumor* OR tumor*)).ti,ab 171
- 70 CINAHL (neurohypophysial AND neoplasm*).ti,ab 0
- 71 CINAHL (oligoastrocytoma).ti,ab 45
- 72 CINAHL (oligoastrocytic AND (tumor* OR tumor*)).ti,ab 1
- 73 CINAHL (oligodendroglioma).ti,ab 112
- 74 CINAHL (oligodendrocytosis).ti,ab 0
- 75 CINAHL (parenchymal AND (tumor* OR tumor*)).ti,ab 102
- 76 CINAHL ("pilocytic astrocytoma").ti,ab 26
- 77 CINAHL ("pineal gland" AND (tumor* OR tumor*)).ti,ab 15
- 78 CINAHL (pineoblastoma).ti,ab 5
- 79 CINAHL PINEALOMA/ 29
- 80 CINAHL "INFRATENTORIAL NEOPLASMS"/ 73
- 81 CINAHL (pinealoma).ti,ab 2
- 82 CINAHL (pineocytoma).ti,ab 3
- 83 CINAHL (PNET).ti,ab 100
- 84 CINAHL ("primitive neuroectodermal" AND (tumor* OR tumor*)).ti,ab 135
- 85 CINAHL ("pons angle" AND (tumor* OR tumor*)).ti,ab 1
- 86 CINAHL ("pontine neoplasm").ti,ab 0
- 87 CINAHL (pontine AND (tumor* OR tumor)).ti,ab 34
- 88 CINAHL (pontine AND glioma).ti,ab 25
- 89 CINAHL ("posterior fossa" AND neoplasm*).ti,ab 10
- 90 CINAHL ("posterior fossa" AND (tumor* OR tumor*)).ti,ab 108
- 91 CINAHL (spongioblastoma).ti,ab 1
- 92 CINAHL "NEOPLASMS, NEUROEPITHELIAL"/ 81
- 93 CINAHL ((subtentorial AND tumor*) AND tumor*).ti,ab 0
- 94 CINAHL ("supratentorial neoplasm*").ti,ab 2
- 95 CINAHL (tentorial AND meningioma).ti,ab 1
- 96 CINAHL MENINGIOMA/ 493
- 97 CINAHL (cerebri AND (tumor* OR tumor*)).ti,ab 20
- 98 CINAHL (ventrical AND (tumor* OR tumor*)).ti,ab 0
- 99 CINAHL (ventricular AND (tumor* OR tumor*)).ti,ab 294
- 100 CINAHL (13 OR 14 OR 15 OR 16 OR 17 OR 18 OR 19 OR 20 OR 21 OR 22 OR 23 OR 24 OR 25 OR 26 OR 27 OR 28 OR 29 OR 30 OR 31 OR 32 OR 33 OR 34 OR 35 OR 36 OR 37 OR 38 OR 39 OR 40 OR 41 OR 42 OR 43 OR 44 OR 45 OR 46 OR 47 OR 48 OR 49 OR 50 OR 51 OR 52 OR 53 OR 54 OR 55 OR 56 OR 57 OR 58 OR 59 OR 60 OR 61 OR 62 OR 63 OR 64 OR 65 OR 66 OR 67 OR 68 OR 69 OR 70 OR 71 OR 72 OR 73 OR 74 OR 75 OR 76 OR 77 OR 78 OR 79 OR 80 OR 81 OR 82 OR 83 OR 84 OR 85 OR 86 OR 87 OR 88 OR 89 OR 90 OR 91 OR 92 OR 93 OR 94 OR 95 OR 96 OR 97 OR 98 OR 99) 9685.
- 101 CINAHL (animal* OR "animal health" OR "animal population" OR "animal research" OR "animal study" OR "animal studies" OR "laboratory animal" OR primate OR rabbit OR rodent OR rat).ti,ab 44,880.
- 102 CINAHL "ANIMALS, LABORATORY"/ OR "ANIMAL POPULATION GROUPS"/ OR ANIMALS/35299
- 103 CINAHL "ANIMAL STUDIES"/ 43,675
- 104 CINAHL (101 OR 102 OR 103) 90,301
- 105 CINAHL (12 AND 100) 78
- 106 CINAHL (103 AND 105) 11
- 107 CINAHL (102 AND 105) 12

## Appendix B-MeSH terms

- Brain edema
- Brain hematoma
- Brain edema
- Brain swelling
- Cerebral edema
- Cerebral edema
- Cerebral swelling
- Cytotoxic brain edema
- Cytotoxic cerebral edema
- Intracranial edema
- Vasogenic brain edema
- Vasogenic cerebral edema
- Brain Disease, Edema (< 1992)
- Astrocytoma
- Astroglioma
- Brain cancer
- Brain tumor(s)
- Brain tumor(s)
- Brain Tumor Metastasis
- Brain neoplasm(s)
- Brain neoplasm(s), benign
- Brain stem tumor(s)
- Brain stem tumor(s)
- Brain tumor, malignant
- Brain tumor, malignant
- Brain tumor, primary
- Brain tumor, primary
- Brain tumor, recurrent
- Brain tumor, recurrent
- Cerebellar Neoplasm(s)
- Cerebellar Tumor(s)
- Cerebellar Tumor(s)
- Cerebellum tumor
- Cerebral Astrocytoma
- Cerebral Ventricle Tumor(s)
- Cerebral Ventricle Tumor(s)
- Cerebral Ventricle Neoplasm(s)
- Cerebroventricular Neoplasm(s)
- Cerebello Pontine Angle Tumor
- Choroid Plexus Neoplasms
- Choroid Plexus Tumors
- Ependymoma
- Ependymoblastoma
- Fibrillary Astrocytoma
- Ganglioglioma
- Gemistocytic Astrocytoma
- Glioma
- Glioma, Astrocytic
- Glioma, Mixed
- Glioma, Subependymal
- Glioblastoma, Giant Cell
- Gliosarcoma
- Glial Cell Tumor(s)
- Glial Cell Tumor(s)
- Hypothalamic Cancer
- Hypothalamic Neoplasm(s)
- Hypothalamic Teratomas
- Hypothalamic Tumor(s)
- Hypothalamic Tumor(s)
- hypothalamus tumor
- Hypophysis tumor
- Infratentorial Cancer
- Infratentorial Neoplasm(s)
- Infratentorial Tumor(s)
- Infratentorial Tumor(s)
- Intracranial Astrocytoma
- Intracranial Neoplasm(s)
- Malignant Glioma
- Medullary Neoplasm(s)
- Medullary Tumor(s)
- Medullary Tumor(s)
- Medulloepithelioma
- Mesencephalic Neoplasm(s)
- Midbrain Tumor(s)
- Midbrain Tumor(s)
- Myxopapillary Ependymoma
- Neoplasm Metastases
- Neoplasm Micrometastases
- Neurocytoma
- Neuroectodermal Tumors, Primitive
- Neurohypophysial Region Neoplasms
- Oligoastrocytoma.
- Oligoastrocytic tumors
- Oligodendroglioma
- Oligodendrocytosis
- Parenchymal Tumor
- Pilocytic Astrocytoma
- Pineal Gland Tumor
- Pineal Gland Tumor
- Pineal Tumor(s)
- Pineal Tumor(s)
- Pineoblastoma
- Pinealoma
- Pineocytoma
- Pineocytoma-Pineoblastoma
- PNET
- Pons angle tumor
- Pontine Neoplasm(s)
- Pontine Tumor(s)
- Pontine Tumor(s)
- Pontine Glioma
- Posterior Fossa Neoplasm(s)
- Posterior Fossa Tumor
- Posterior Fossa Tumor
- Primitive Neuroepithelial Neoplasms
- Spongioblastoma
- Subependymoma
- Subtentorial Tumor
- Supratentorial Neoplasm(s)
- Tentorial Meningioma
- Tentorium Meningioma
- Tumor, Cerebri
- Ventricle Tumor, Brain
- Ventricular Tumors, Brain
- Animal(s)
- Animal health
- Animal population
- Animal research
- Animal studies
- Laboratory animals
- Non-human data
- Primates
- Rabbits
- Rats
- Rodents
- Animal v Human comparative study

## Data Availability

The datasets generated during and/or analysed during the current study are available from the corresponding author on reasonable request.
